# SARS-CoV-2 mutations, vaccines, and immunity: implication of variants of concern

**DOI:** 10.1038/s41392-021-00623-2

**Published:** 2021-05-22

**Authors:** Ji Yun Noh, Hye Won Jeong, Eui-Cheol Shin

**Affiliations:** 1 Division of Infectious Diseases, Department of Internal Medicine, Korea University Guro Hospital, Korea University College of Medicine, Seoul, Republic of Korea; 2 Graduate School of Medical Science and Engineering, Korea Advanced Institute of Science and Technology (KAIST), Daejeon, Republic of Korea; 3 Division of Infectious Diseases, Department of Internal Medicine, Chungbuk National University College of Medicine, Cheongju, Republic of Korea; 4 The Center for Epidemic Preparedness, KAIST, Daejeon, Republic of Korea

**Keywords:** Vaccines, Infectious diseases

In a recent study published in *Nature*, Wang et al.^1^ investigated the neutralizing activities of antibodies elicited by COVID-19 mRNA vaccines and natural infection with SARS-CoV-2 against SARS-CoV-2 variants.

The devastating impact of the ongoing COVID-19 pandemic on public health, the economy, and society has made vaccine development a top priority for global health. Thus, vaccine development is progressing at an unprecedented pace as an urgent response to COVID-19.

Currently, there are four main types of COVID-19 vaccine: nucleic acid (mRNA and DNA), viral vector, protein subunit, and inactivated virus. Two COVID-19 mRNA vaccines (BNT162b2 developed by Pfizer-BioNTech and mRNA-1273 by Moderna) have been authorized by the U.S. Food and Drug Administration (FDA) and European Medicines Agency (EMA). In addition, Ad26.COV2.S (Johnson & Johnson/Janssen) was approved by the FDA and EMA and ChAdOx1 nCoV-19 (AstraZeneca) was authorized by the EMA, both of which are viral vector vaccines.

BNT162b2 and mRNA-1273 are lipid nanoparticle-formulated, nucleoside-modified RNA vaccines encoding the prefusion spike glycoprotein of SARS-CoV-2. Both of them have shown favorable vaccine efficacy (94–95%) in preventing COVID-19 in phase 3 clinical trials. However, emerging variants of SARS-CoV-2 and its global expansion have raised concerns about potentially reduced protection against variants of concern (VOCs) by current COVID-19 vaccines. Notable variants harboring multiple mutations in the spike protein have emerged in the United Kingdom (B.1.1.7), South Africa (B.1.351), and Brazil (P.1). B.1.1.7 variant (20I/501Y.V1), the most globally widespread VOC, has a N501Y substitution in the receptor-binding domain (RBD), H69/V70 deletion in the N-terminal domain, and P681H mutation adjacent to the furin cleavage site in the spike protein. This variant has been associated with increased transmissibility. B.1.351 variant (20H/501Y.V2) contains several mutations, including K417N, E484K, and N501Y. P.1 variant (B.1.1.28.1) possesses K417T, E484K, and N501Y substitution in the RBD of the spike protein. These VOCs also share the D614G mutation, which confers an increased ability for rapid viral spread.

Wang et al.^1^ tested the neutralizing activity of plasma from vaccinees (BNT162b2, *n* = 6; mRNA-1273, *n* = 14) against pseudotype viruses harboring K417N, E484K, N501Y, and a combination of these three RBD mutations (B.1.351 variant). The study revealed 1- to 3-fold decreased neutralizing activity against E484K, N501Y, and the K417N:E484K:N501Y combination (*p* = 0.0033, *p* = 0.0002, and *p* < 0.0001, respectively), but there was no significant difference in neutralizing activity against wild-type and K417N mutation. This result suggests that COVID-19 mRNA vaccine-elicited neutralizing antibodies are less effective against emerging SARS-CoV-2 VOCs with RBD mutations. In addition, convalescent plasma obtained 6 months after SARS-CoV-2 infection was 0.5- to 20.2-fold less effective in neutralizing the K417N:E484K:N501Y combination (*p* < 0.0001).

In a subsequent analysis, the study was extended to SARS-CoV-2 RBD-specific memory B cells. The mRNA vaccines elicited a robust SARS-CoV-2 RBD-specific memory B-cell response similar to natural infection. Monoclonal antibodies (mAbs) were expressed by SARS-CoV-2 RBD-specific memory B cells and had potent neutralizing activity towards SARS-CoV-2 pseudovirus. However, among the 17 most potent mAbs, 14 demonstrated reduced or abolished activities in neutralizing the K417N, E484K, or N501Y mutations. Selection pressure by mAbs was also detected; these mutations emerged when recombinant vesicular stomatitis virus/SARS-CoV-2 S was cultured in the presence of the vaccine-elicited mAbs.

Similarly, Chen et al.^2^ reported that sera from BNT162b2-vaccinated individuals showed reduced neutralizing activities against emerging SARS-CoV-2 variants. They observed significantly decreased neutralizing potency of sera from the vaccinees against B.1.1.7 isolate (2-fold), E484K/N501Y/D614G recombinant variant (4-fold), and two chimeric SARS-CoV-2 strains encoding B.1.351 spike (10-fold) and P.1 spike (2.2-fold) compared to the D614G variant in Vero-hACE2-TMPRSS2 cells. A notable reduction in neutralizing activity was not shown with the K417N/D614G variant, suggesting that sera from recipients of the BNT162b2 vaccine had a lower neutralizing capacity against E484K and N501Y-containing viruses.

In addition, Wang et al.^3^ demonstrated a substantial loss of neutralizing activity against the B.1.351 strain in convalescent plasma (9.4-fold) and sera from vaccinees who received mRNA vaccines (10.3–12.4-fold). E484K seemed to be the main contributor to the neutralization resistance. Furthermore, the neutralizing potency of mAb therapeutics in clinical use or under clinical investigation decreased against the B.1.351 variant.

**Fig. 1:**
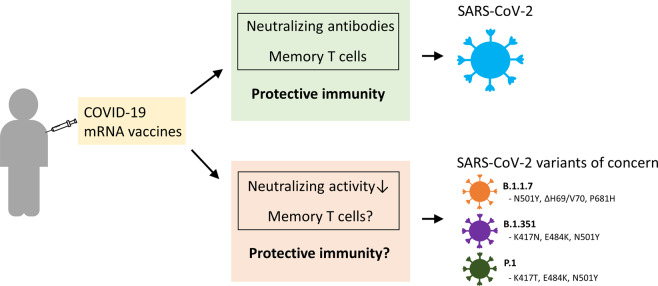
COVID-19 vaccines elicit SARS-CoV-2-specific CD4^+^ and CD8^+^ T-cell responses as well as neutralizing antibodies. Even though antibodies elicited by current COVID-19 mRNA vaccines had shown diminished neutralizing activities against SARS-CoV-2 variants, T-cell responses may have a role for host protection against SARS-CoV-2 variants

Taken together, the results from recent studies suggest that the emergence of resistant SARS-CoV-2 variants may nullify the effects of current COVID-19 vaccines. However, COVID-19 vaccines can elicit not only neutralizing antibodies, but also SARS-CoV-2-specific CD4^+^ and CD8^+^ T-cell responses. Vaccination with various vaccine platforms, including mRNA and viral vectors, has been shown to elicit SARS-CoV-2-specific CD4^+^ and CD8^+^ T-cell responses (Fig. 1). In principle, it is more difficult to evade T-cell responses than a neutralizing antibody response because multiple T-cell epitopes are scattered across viral proteins, whereas neutralizing antibody targets a narrow region in the viral protein. Although SARS-CoV-2 mutations that abrogate binding to major histocompatibility complex have been reported,^4^ Tarke et al.^5^ recently reported an insignificant impact of SARS-CoV-2 variants on both CD4^+^ and CD8^+^ T-cell responses in COVID-19 convalescents and recipients of COVID-19 mRNA vaccines. T-cell responses to the variants B.1.1.7, B.1.351, P.1, and CAL.20C (emerged in Southern California) were not differ from those to the ancestral strain of SARS-CoV-2. Most SARS-CoV-2 T-cell epitopes were conserved despite the mutations in the variants.

The future of the current COVID-19 pandemic is unpredictable. Careful surveillance for the emergence of variants and a thorough investigation of its impact on public health will be continuously required. Continuous emergence of SARS-CoV-2 VOCs have implications for updating current COVID-19 vaccines and the development of vaccines providing broader protection. Even if SARS-CoV-2 variants escape the neutralizing antibodies elicited by current COVID-19 vaccines, T-cell immunity may be helpful in reducing the disease burden of COVID-19 by attenuating disease severity and decreasing mortality. The real-world effectiveness of the COVID-19 vaccines, especially in preventing hospitalization, complications, and death, should be assessed in the near future. Above all, public health policies should be implemented to ensure that concerns about SARS-CoV-2 variants and possible reduced vaccine efficacy towards VOCs do not lead to blunted COVID-19 vaccination rates.
